# Assessing subgroup effects with binary data: can the use of different effect measures lead to different conclusions?

**DOI:** 10.1186/1471-2288-5-15

**Published:** 2005-04-29

**Authors:** Ian R White, Diana Elbourne

**Affiliations:** 1 MRC Biostatistics Unit Institute of Public Health, Robinson Way, Cambridge CB2 2SR, UK; 2 Medical Statistics Unit London School of Hygiene and Tropical Medicine, Keppel Street, London WC1E 7HT, UK

## Abstract

**Background:**

In order to use the results of a randomised trial, it is necessary to understand whether the overall observed benefit or harm applies to all individuals, or whether some subgroups receive more benefit or harm than others. This decision is commonly guided by a statistical test for interaction. However, with binary outcomes, different effect measures yield different interaction tests. For example, the UK Hip trial explored the impact of ultrasound of infants with suspected hip dysplasia on the occurrence of subsequent hip treatment. Risk ratios were similar between subgroups defined by level of clinical suspicion (P = 0.14), but odds ratios and risk differences differed strongly between subgroups (P < 0.001).

**Discussion:**

Interaction tests on different effect measures differ because they test different null hypotheses. A graphical technique demonstrates that the difference arises when the subgroup risks differ markedly. We consider that the test of interaction acts as a check on the applicability of the trial results to all included subgroups. The test of interaction should therefore be applied to the effect measure which is least likely *a priori *to exhibit an interaction. We give examples of how this might be done.

**Summary:**

The choice of interaction test is especially important when the risk of a binary outcome varies widely between subgroups. The interaction test should be pre-specified and should be guided by clinical knowledge.

## Background

### Subgroup analysis

Any randomised controlled trial, however tight its inclusion and exclusion criteria, recruits individuals who differ in many observed and unobserved ways. Different individuals are rarely likely to respond to intervention in exactly the same way, so the intervention effect within carefully defined subgroups is of interest. Unfortunately, analysis of a trial within subgroups is usually underpowered: results often have wide confidence intervals and lack statistical significance, even when the trial intervention is beneficial. Further, repeating an analysis within several subgroups greatly increases the risk of false positive findings [1]. Subgroup analysis must therefore be treated cautiously, and "the overall trial result is usually a better guide to the direction of effect in subgroups than the apparent effect observed within a subgroup" [2].

When a trial outcome is binary, a number of different effect measures are available [3]: the risk difference or absolute benefit, which is easily translated into the clinically relevant number needed to treat [4]; the risk ratio, which is widely understood [5]; or the odds ratio, which has desirable statistical properties [6,7]. Further, the risk ratio for benefits gained differs from the risk ratio for harm avoided. Subgroups that are identical on one of these effect measures are not usually identical on a different effect measure. For example, if intervention halves the risk in each subgroup, and the subgroups themselves have different risks, then the risk differences differ between subgroups.

When subgroups have different risks, it is common to estimate an overall risk ratio and then, using the control group risk in each subgroup, to infer the subgroup-specific risk difference and number needed to treat [8]. In this approach, the estimated absolute benefit of intervention is proportional to the control group risk. However, this assumes the risk ratio is equal across subgroups. In making a treatment decision about a particular patient, therefore, the clinician makes best use of the evidence base by ignoring possible differences in the chosen measure of treatment effect between subgroups. It is important to have tools to indicate when this is inappropriate.

### Interaction

The statistical test of interaction is a useful tool in this dilemma. In statistical language, interaction is the difference between the intervention effects in different subgroups, and the null hypothesis is that the intervention effect is equal across subgroups [9-12]. A statistically significant interaction supports placing more weight on subgroup-specific findings, especially if it arises from one of a small number of pre-defined subgroup analyses. On the other hand, a non-significant interaction suggests that the overall trial findings should inform individual intervention decisions. The clinical plausibility and importance of the subgroup-specific findings must also be taken into account [13].

It is useful to distinguish a qualitative interaction, in which intervention is beneficial in one subgroup but ineffective or harmful in another, from a quantitative interaction in which intervention is beneficial in all subgroups (or harmful in all) but the degree of benefit varies [14]. As an example of quantitative interaction, a large meta-analysis showed that tamoxifen has benefit in treating both oestrogen-receptor-positive and oestrogen-receptor-negative early breast cancer, but that the benefit is greater in the first group [15]. Such clear-cut statistically significant results are rare in single trials.

When the trial outcome is binary, discussion of interactions is further complicated by the variety of possible effect measures. Significance tests on the overall intervention effect are unaffected by the choice of measure, but the existence and strength of interactions depend on the effect measure used [16]. Quantitative interactions can usually be removed by changing the effect measure, but qualitative interactions cannot be removed in this way.

### UK Hip trial

We explore these issues in the context of the UK Hip trial [17]. This trial aimed to show that diagnostic ultrasound in the management of infants with suspected developmental hip dysplasia reduced overall treatment (mainly splinting) without doubling the risk of operative treatment. Note that treatment is an outcome in this trial. Results were reported as risk ratios for operative treatment comparing ultrasound with no ultrasound. The observed risks were 21/314 (7%) and 25/315 (8%) respectively, so the overall risk ratio was 0.84 with a 95% confidence interval from 0.48 to 1.47, suggesting that risk of operative treatment was not doubled. In Table 1 this risk ratio for operative treatment is termed "risk ratio for harm".

**Table 1:** Operative treatment in the UK Hip trial

Level of clinical suspicion	Ultrasound	No Ultrasound	Risk ratio for harm (95% CI)	Risk ratio for benefit (95% CI)	Odds ratio (95% CI)	Risk difference (95% CI)
All	21/314 (7%)	25/315 (8%)	0.84 (0.48 to 1.47)	1.01 (0.97 to 1.06)	0.83 (0.46 to 1.52)	-0.01 (-0.05 to 0.03)

Strong	7/95 (7%)	11/89 (12%)	0.60 (0.24 to 1.47)	1.06 (0.96 to 1.16)	0.56 (0.21 to 1.53)	-0.05 (-0.14 to 0.04)
Moderate	14/219 (6%)	14/226 (6%)	1.03 (0.50 to 2.11)	1.00 (0.95 to 1.05)	1.03 (0.48 to 2.22)	0.00 (-0.04 to 0.05)

Test of interaction^1^			P = 0.35	P = 0.29	P = 0.34	P = 0.30

^1 ^Mantel-Haenszel test.

Infants fell into two subgroups defined by level of clinical suspicion prior to randomisation: strong suspicion, defined as "sufficient to warrant early prophylactic splinting", or moderate suspicion, defined as "sufficient to warrant further specialist examination". The risk ratio for the moderate-suspicion subgroup (Table 1) has a 95% confidence interval that includes a doubling of the risk of operative treatment. However, the risk ratios are not significantly different on an interaction test (P = 0.35). This suggests using the overall relative risk of 0.84, with its upper confidence limit of 1.47, as applying to both groups.

Alternative analyses based on the other effect measures are shown in the last three columns of Table 1. The risk ratios for benefit (the risk ratios for avoiding operative treatment) are all near 1, because the outcome event is rare. For the same reason, the odds ratio is numerically similar to the risk ratio. The risk difference is numerically very different. However, all four effect measures give very similar significance levels on the interaction test.

A second outcome measure in the UK Hip trial was the occurrence of any hip treatment (Table 2). Clinical suspicion is a strong prognostic factor for this outcome: in the no-ultrasound arm, 97% of the strong-suspicion group but only 32% of the moderate-suspicion group received hip treatment. The risk ratios, risk differences and odds ratios all show a larger effect in the strong-suspicion subgroup. However, the risk ratios for harm do not differ significantly between subgroups, yet the risk ratios for benefit, the risk differences and the odds ratios all have highly statistically significant differences between subgroups.

**Table 2:** Any hip treatment in the UK Hip trial

Level of clinical suspicion	Ultrasound	No Ultrasound	Risk ratio for harm (95% CI)	Risk ratio for benefit (95% CI)	Odds ratio (95% CI)	Risk difference (95% CI)
All	126/314 (40%)	159/315 (50%)	0.79 (0.67 to 0.95)	1.21 (1.05 to 1.40)	0.66 (0.48 to 0.90)	-0.10 (-0.18 to -0.03)

Strong	65/95 (68%)	86/89 (97%)	0.71 (0.61 to 0.82)	9.37 (2.96 to 29.62)	0.08 (0.02 to 0.24)	-0.28 (-0.38 to -0.18)
Moderate	61/219 (28%)	73/226 (32%)	0.86 (0.65 to 1.15)	1.07 (0.94 to 1.20)	0.81 (0.54 to 1.21)	-0.04 (-0.13 to 0.04)

Test of interaction^1^			P = 0.14	P < 0.001	P < 0.001	P < 0.001

^1 ^Mantel-Haenszel test.

## Discussion

### Why do interaction tests differ?

Interaction tests on different scales differ because they are testing different null hypotheses. In the UK Hip trial, ultrasound reduced the risk of any hip treatment from 97% to 68% in the strong-suspicion group. Under the null hypothesis of a common risk ratio for hip treatment, the 32% risk in the moderate-suspicion group would be reduced to 23%, but the null hypothesis of a common risk difference implies a reduction to 4.1%, and the null hypothesis of a common odds ratio implies a reduction to 3.5%. The observed reduction to 28% is consistent only with the null hypothesis of a common risk ratio. A common risk ratio for avoiding hip treatment is not possible, since the risk ratio is over 9 in the strong-suspicion group, and multiplying the 68% risk of avoiding hip treatment in the strong-suspicion group by 9 would result in a risk over 100%.

### When do interaction tests differ markedly?

**Figure 1:**
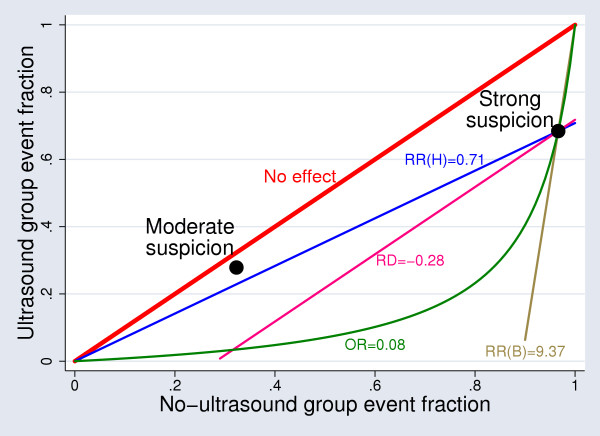
UK Hip trial: results for any treatment in strong-suspicion and moderate-suspicion subgroups. Points on the red diagonal line indicate lack of effect; points on the other lines indicate the same risk difference (RD), risk ratio for harm (RR(H)), risk ratio for benefit (RR(B)) or odds ratio (OR) as in the strong-suspicion subgroup.

A graph of the event fraction in the ultrasound group against the event fraction in the no-ultrasound group is helpful (Figure 1) [18]. Points below the diagonal line indicate a lower event fraction in the ultrasound group. The large dots represent the results for the two subgroups. The curved line shows results that have the same odds ratio as the strong-suspicion subgroup, while other lines show results that have the same risk difference or risk ratio. The moderate-suspicion subgroup lies closest to the line of a common risk ratio for harm. It is clear from Figure 1 that the choice of effect measure matters most when the subgroup risks differ markedly.

### Which interaction test is best?

Since the four interaction tests may differ markedly, it is important to make a careful choice. An intuitive approach is to perform the interaction analysis on the same scale on which the results are to be presented [19]. For example, clinical trial results are often presented on the risk ratio for harm scale, so the interaction analysis would test equality of these risk ratios. However, there are other considerations.

Deeks discussed selection of an effect measure or summary statistic in meta-analysis [16]. His aim was to find a summary statistic that is most plausibly equal in all trials, including those with different control group risks, in order to best predict the treatment benefit for various patient types. One way is to select an effect measure for which the subgroup-specific results are comparable, as judged by the interaction test. In meta-analysis, the effect measure minimising the Q (heterogeneity) statistic could be used. Using this approach in the UK Hip trial, the results for any hip treatment would have been reported on the risk ratio for harm scale, regardless of what had been planned. However, Deeks argues that this is problematic with the typically small number of trials in a meta-analysis. Instead, the choice of effect measure should use both clinical knowledge and empirical evidence. For example, given the clinical view that absolute benefit is likely to be greatest in those with greatest risk, the risk ratio for harm would appear the best effect measure. Empirically, Deeks shows that the risk ratio for harm and the odds ratio are more frequently homogeneous between trials than the risk ratio for benefit and the risk difference, supporting their wider use.

Deeks' arguments apply to meta-analysis, and we would not apply them to subgroup analysis within clinical trials. Instead, we view interaction tests as a check on the applicability of the trial results to all included subgroups. Investigators start with the belief that all subgroups recruited to the trial have qualitatively similar responses to intervention. It is reasonable to maintain that belief if it can be shown to be consistent with the data. We therefore propose that investigators should identify the effect measure that is most likely to be similar between subgroups. By carefully specifying this measure in advance, they ensure that the interaction test has maximum scientific validity.

For example, suppose that the investigators designing the UK Hip trial had predicted that 95% and 30% of the two subgroups would receive treatment in the absence of ultrasound. They could then have asked: if ultrasound reduced the proportion in the first subgroup from 95% to 70%, what effect is likely in the second subgroup? A reduction from 95% to 70% represents a risk ratio for harm of 0.74, which would reduce 30% to 22% in the second subgroup. A reduction from 95% to 70% also represents an odds ratio of 0.12 and a risk difference of 25 percentage points, both of which (by coincidence) would reduce 30% to 5% in the second subgroup. A common risk ratio for benefit is impossible with these numbers, as noted above. The choice among these possibilities will draw on investigators' knowledge and experience. If the investigators believed that all infants are equally able to be saved from treatment, then a common risk ratio for harm would be plausible. If on the other hand the lower treatment rate in the second subgroup implies less pathology and hence greater potential for avoiding treatment, then a common odds ratio might be more plausible.

As another example, consider a trial of a community intervention to promote vaccination. Suppose that the expected unvaccinated fractions in two subgroups are 20% and 80% without the intervention, and that the intervention is expected to halve the unvaccinated fraction in the first subgroup. If the difference between subgroups stems from a lack of previous vaccination campaigns, then all unvaccinated individuals would be equally likely to be vaccinated under the intervention, so the second subgroup would see a reduction to 40% – a common risk ratio of 0.5. But if the difference between subgroups stems from the second subgroup's greater suspicion of vaccination, then their likely reduction would be smaller – perhaps to the figure of 64% which represents a common odds ratio of 0.44.

We would usually prefer primary results to be presented on the scale selected for interaction testing. While it may be appropriate to present subgroup-specific results on a different scale, it would not be correct to use that scale for a single summary measure over the whole trial. For example, subgroups with equal odds ratios usually have unequal risk ratios, so a single summary risk ratio as proposed by Zheng and Yu [20] would generally be inappropriate. Just as a single risk ratio may be used to compute separate risk differences or numbers needed to treat, so a common odds ratio could be used to compute fitted risks in all subgroups and hence to compute appropriate risk ratios, risk differences or numbers needed to treat.

Finally, the choice of effect measure for the interaction test is important because it may affect conclusions about the applicability of the overall results of a clinical trial to all subgroups. This choice should therefore be specified in advance of data analysis. The best place to do this is in a trial protocol or statistical analysis plan.

## Conclusion

A statistical test of interaction is important in deciding whether the overall results of a randomised trial apply to all subgroups. When the outcome is binary, different effect measures may lead to very different results on the test of interaction. The choice of effect measure for the test of interaction should therefore be specified before analysis of the data. The best choice of effect measure for the test of interaction is that which the investigators believe is most likely to be similar between subgroups.

## Competing interests

DE received funding from the Department of Health, via the Medical Research Council, for the UK Hip Trial [17]. Apart from this, neither author has a competing interest.

## Authors' contributions

The original idea arose in discussion between the authors. IRW wrote the first draft. Both authors contributed to subsequent drafts and approved the final version.

## Pre-publication history

The pre-publication history for this paper can be accessed here:


